# Strategic use of new generation antidepressants for depression: SUN(^_^) D protocol update and statistical analysis plan

**DOI:** 10.1186/s13063-015-0985-6

**Published:** 2015-10-14

**Authors:** Naohiro Yonemoto, Shiro Tanaka, Toshi A. Furukawa, Tadashi Kato, Akio Mantani, Yusuke Ogawa, Aran Tajika, Nozomi Takeshima, Yu Hayasaka, Kiyomi Shinohara, Kazuhira Miki, Masatoshi Inagaki, Shinji Shimodera, Tatsuo Akechi, Mitsuhiko Yamada, Norio Watanabe, Gordon H. Guyatt, for the SUN(^_^)D Investigators

**Affiliations:** Department of Neuropsychopharmacology, National Institute of Mental Health, National Center of Neurology and Psychiatry, 4-1-1 Ogawa-Higashi, Kodaira, Tokyo 187-8553 Japan; Leuven Biostatistics and Statistical Bioinformatics Center, Katholic University of Leuven, Kapucijnenvoer 35, 3000 Leuven, Belgium; Department of Pharmacoepidemiology, Kyoto University Graduate School of Medicine/of Public Health, Yoshida Konoe-cho, Sakyo-ku, Kyoto 606-8501 Japan; Department of Health Promotion and Human Behavior, Kyoto University Graduate School of Medicine/School of Public Health, Yoshida Konoe-cho, Sakyo-ku, Kyoto 606-8501 Japan; Department of Clinical Epidemiology, Kyoto University Graduate School of Medicine/School of Public Health, Yoshida Konoe-cho, Sakyo-ku, Kyoto 606-8501 Japan; Aratama Mental Clinic, 1-49 Suyama-cho, Mizuho-ku, Nagoya 467-0066 Japan; Mantani Mental Clinic, 5-18 Itsukaichi Ekimae 1-chome, Saeki-ku, Hiroshima 731-5125 Japan; Miki Mental Clinic, 1-3 Hiranuma 1-chome, Nishi-ku, Yokohama 220-0023 Japan; Department of Neuropsychiatry, Okayama University Hospital, 2-5-1, Shikata-cho, Kita-ku, Okayama 700-8558 Japan; Department of Neuropsychiatry, Kochi Medical School, Kohasu, Okoh-cho, Nankoku, 783-8505 Japan; Department of Psychiatry and Cognitive-Behavioral Medicine, Nagoya City University Graduate School of Medical Sciences, Mizuho-cho, Mizuho-ku, Nagoya, 467-8601 Japan; Translational Medical Center, National Center of Mental Health, National Center of Neurology and Psychiatry, 4-1-1 Ogawa-Higashi, Kodaira, Tokyo 187-8502 Japan; Department of Clinical Epidemiology and Biostatistics, McMaster University, West Hamilton, Ontario L8N 3Z5 Canada

## Abstract

**Background:**

SUN(^_^)D, the Strategic Use of New generation antidepressants for Depression, is an assessor-blinded, parallel-group, multicenter pragmatic mega-trial to examine the optimum treatment strategy for the first- and second-line treatments for unipolar major depressive episodes. The trial has three steps and two randomizations. Step I randomization compares the minimum and the maximum dosing strategy for the first-line antidepressant. Step II randomization compares the continuation, augmentation or switching strategy for the second-line antidepressant treatment. Step III is a naturalistic continuation phase. The original protocol was published in 2011, and we hereby report its updated protocol including the statistical analysis plan.

**Results:**

We implemented two important changes to the original protocol. One is about the required sample size, reflecting the smaller number of dropouts than had been expected. Another is in the organization of the primary and secondary outcomes in order to make the report of the main trial results as pertinent and interpretable as possible for clinical practices. Due to the complexity of the trial, we plan to report the main results in two separate reports, and this updated protocol and the statistical analysis plan have laid out respective primary and secondary outcomes and their analyses. We will convene the blind interpretation committee before the randomization code is broken.

**Conclusion:**

This paper presents the updated protocol and the detailed statistical analysis plan for the SUN(^_^)D trial in order to avoid reporting bias and data-driven results.

**Trial registration:**

ClinicalTrials.gov: NCT01109693 (registered on 21 April 2010).

## Update

This paper provides the updated protocol and the detailed statistical analysis plan for the Strategic Use of New generation antidepressants for Depression, SUN (^_^)D, randomized controlled trial, a pragmatic mega-trial examining the optimum treatment strategy for the first- and second-line treatments for unipolar major depressive episodes. The original protocol was published in *Trials* in 2011 [1], and we hereby report its updated protocol including the statistical analysis plan, as we would like to implement two important changes to the original protocol. One change is about the required sample size, reflecting the smaller number of dropouts than had been expected. Another change is in the organization of the primary and secondary outcomes in order to make the report of the main trial results as pertinent and interpretable as possible for clinical practices.

The trial completed recruitment of all participants on 13 March 2015, and completion of the last follow-up is expected in September 2015. This updated protocol and statistical analysis plan were drafted without knowledge of the randomization code, which will not be broken before acceptance of the current paper for publication.

### Trial overview

#### Trial design

**Fig. 1:**
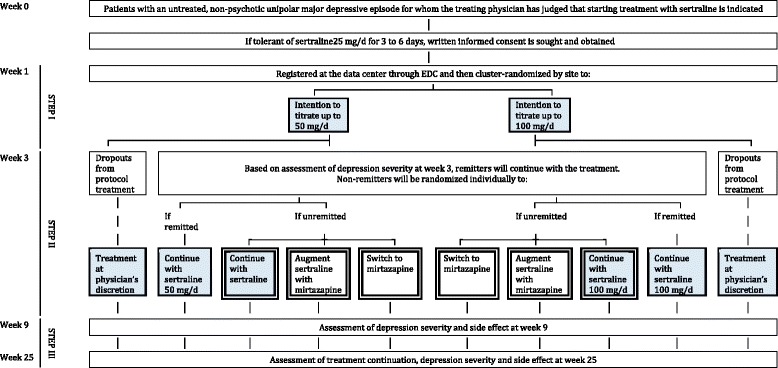
Flow diagram of the trial

SUN(^_^)D is an assessor-blinded, parallel-group, multicenter randomized controlled trial [1]. The trial has three steps with two randomizations (Fig. 1). Randomizations were performed at first recruitment for Step I (cluster randomization by site) and at start of Step II (individual randomization). Step I has two arms, in which sertraline will be started from 25 mg/d and titrated up to 50 mg/d or up to 100 mg/d, the minimum and the maximum of the standard prescription range in Japan, respectively. Step II has three arms, in which sertraline will be continued as in Step I, mirtazapine will be added to sertraline, or sertraline will be switched to mirtazapine. In Step III, all the treatments will be at the discretion of the treating physician.

Treatment of a major depressive episode is commonly divided into the acute phase treatment, which aims at the prompt reduction of acute symptoms, and the continuation treatment, which not only seeks to reduce further the symptoms but also to prevent symptom relapses [2, 3]. The acute phase treatment usually lasts 6 to 12 weeks, and it is recommended that the continuation treatment be continued 4 to 9 months after the acute phase treatment [2, 3]. In our study, therefore, Steps I and II represent the acute phase treatment, whereas Step III corresponds with the continuation treatment.

### Ethics

This study is being conducted in accordance with the Declaration of Helsinki and its amendments as well as the Ethics Guideline for Clinical Research (2008 revision, Ministry of Health, Labor and Welfare, Japan). Written informed consent has been obtained from each participant after full explanation of the purpose and the procedures of the study.

This study has been approved by the Ethics Committee of Kyoto University Graduate School of Medicine (C446), Institutional Review Board of Nagoya City University Hospital (45-10-0004), Ethics Committee of Kochi Medical School (22-96), Ethics Committee of Kumamoto University Graduate School of Life Sciences (Senshin 1341), Institutional Review Board of Yatsushiro Kosei Hospital, Ethics Committee of Yuge Hospital (86), Ethics Committee of Kurume University (11151), Ethics Committee of Saint Lucia Hospital, Ethics Committee of Hiroshima University Hospital (Rin 297), Institutional Review Board of The University of Tokyo Hospital (P2011062-11X), Ethics Committee of Toho University School of Medicine (23053), and Institutional Review Board of Hokkaido University Hospital (Ji 011-0292).

### Funding

This trial was funded by a Grant-in-Aid from the Ministry of Health, Labor and Welfare, Japan (H22-Seishin-Ippan-008) (from April 2010 to March 2012) and by a research project “Pragmatic Psychopharmacotherapy Research” of the Japan Foundation for Neuroscience and Mental Health (from April 2012 to present). The “Pragmatic Psychopharmacotherapy Research” project has received donations from Asahi Kasei Pharma, MSD, Otsuka Pharmaceutical, GlaxoSmithKline, Shionogi, Taisho Pharmaceutical, Mitsubishi Tanabe Pharma, Pfizer, Eli-Lilly, Meiji Seika Pharma, Mochida Pharmaceutical, and Janssen Pharmaceutical as of May 2015.

### Study objectives

As presented in the published protocol [1], the objective of this trial is to establish the optimum first-line and second-line antidepressant treatment strategy among patients with a nonpsychotic unipolar major depressive episode. As the SUN(^_^)D trial involved two randomizations, we will report the main results of the trial as two separate reports.

**REPORT #1** will be entitled “Optimum Target Dose for Initial Sertraline Treatment for Depression: The SUN(^_^)D Trial.”

This report will answer the following clinical question: Which is a superior dosing strategy for the first-line treatment with sertraline, aiming at the lowest or the highest of the standard prescription range, through the acute phase treatment as well as the continuation treatment?

We will compare the following two interventions:

1. Intervention 1: The strategy is to start sertraline at 25 mg/d, with the intention of titrating it up to 50 mg/d where possible by week 3, continuing it up to week 9, and then prescribing at the treating physician’s discretion up to week 25.
2. Intervention 2: The strategy is to start sertraline at 25 mg/d, with the intention of titrating it up to 100 mg/d where possible by week 3, continuing it up to week 9, and then prescribing at treating physician’s discretion up to week 25.

In order to examine the effect of the choice of the initial dosing strategy of the first-line treatment with sertraline through the acute and continuation treatment phases, REPORT #1 will focus on the participants in the blue-shaded cells in Fig. 1.

**REPORT #2** will be entitled “Continue, Switch or Augment after Initial Failure to Remit on First-line Treatment in Depression: The SUN(^_^)D Trial.”

This report will answer the following clinical question: What is the best second-line treatment for patients with no or partial initial response to the first-line treatment with sertraline through the acute phase treatment as well as the continuation treatment?

We will compare intervention 1 with intervention 2, intervention 2 with intervention 3, and intervention 3 with intervention 1 (that is, all combinations without any preferential ordering in importance):

1. Intervention 1: The strategy is to continue sertraline at week 3 and up to week 9, and then prescribing at the treating physician’s discretion up to week 25.
2. Intervention 2: The strategy is to switch sertraline to mirtazapine at week 3 and up to week 9, and then prescribing at the treating physician’s discretion up to week 25.
3. Intervention 3: The strategy is to add mirtazapine to sertraline at week 3 and up to week 9, and then prescribing at the treating physician’s discretion up to week 25.

REPORT #2 will therefore compare the three intervention arms at double borders in Fig. 1 through the acute and continuation treatment phases.

## Outcomes

### Outcome measures

#### Patient health questionnaire-9

The Patient Health Questionnaire-9 (PHQ9) was developed as a self-report version of the Primary Care Evaluation of Mental Disorders (PRIME-MD) [4, 5]. The depression module of the PHQ is called PHQ9 and consists of the nine diagnostic criteria items of the DSM-IV. Each item is rated between 0 = “not at all” through 3 = “nearly every day,” making the total score range from 0 to 27.

Kroenke and his colleagues [6] have provided the following rules of thumb for interpreting the continuous PHQ9 scores:

- 0 to 4 no depression
- 5 to 9 mild depression
- 10 to 14 moderate depression
- 15 to 19 moderately severe depression
- 20 to 27 severe depression

In this trial, PHQ9 and FIBSER will be administered four times (at week 1, week 3, week 9 and week 25) by the blinded central rater via telephone [7].

#### Frequency, intensity, and burden of side effects rating

The Frequency, Intensity, and Burden of Side Effects Rating (FIBSER) was originally used in STAR*D [8, 9] as a global rating scale for side effects. This is an assessor-rated scale, and the Japanese translation has not undergone back translation. Each item is rated in seven grades of severity.

#### Beck depression inventory-II

The Beck Depression Inventory-II (BDI-II) is a 21-item self-report instrument to measure the severity of depression [10, 11]. The time frame for evaluation is set to the past 2 weeks, including the day of assessment. Each item in the BDI-II has a series of four statements, which describe symptom severity along an ordinal continuum from absent or mild (a score of 0) to severe (a score of 3). The total score therefore ranges from 0 through 63.

In this trial, BDI-II will be filled in by the patient at each visit (unblinded).

#### Columbia classification algorithm for suicide assessment

The Columbia Classification Algorithm for Suicide Assessment (C-CASA) is a classification system that utilizes definitions of suicidality [12]. The C-CASA has eight categories that distinguish suicidal events from nonsuicidal events and indeterminate or potentially suicidal events. The scoring is a binary (yes or no) for each item.

### Serious adverse events

A serious adverse event is defined here as “an adverse event that may lead to death or to enduring severe impairment depending on the patient’s conditions and circumstances” and will include the following:

1. Death
2. Life-threatening event
3. Hospitalization or the prolongation of hospitalization
4. Event leading to enduring and severe impairment and dysfunction
5. Congenital malformation

The scoring is a binary yes or no for each item.

### Allowance of timing of outcome assessments

Assessments at week 3, 9 and 25 may be made within the following time frames after week 1:

1. ± 4 days for assessments at weeks 3 through 9
2. ± 14 days for assessments after week 9

If the allowance is violated, we will report it as a deviation from protocol.

### Primary outcome

We conducted two randomizations in this trial, and will therefore make two corresponding reports, each of which will have its own primary outcome for the acute phase treatment as follows:

- **REPORT #1**: Change in PHQ9 from 1 through 9 weeks
- **REPORT #2**: Change in PHQ9 from 3 through 9 weeks

### Secondary outcomes

Secondary outcomes for REPORTS #1 and #2 are summarized below from the viewpoint of the acute phase treatment (up to 9 weeks) and that of the acute through continuation treatments (up to 25 weeks).

**REPORT #1**

Report #1 will include the following:

1. For the acute phase treatment
  (as an index of effectiveness)
    1-1. Change in BDI-II from 1 through 9 weeks
    1-2. Proportion of response (50 % or greater reduction from week 1 in PHQ9) at week 3 and week 9
    1-3. Proportion of remission (4 or less on PHQ9) at week 3 and week 9
  (as an index of acceptability and safety)
    1-4. Proportion of successful continuation of the allocated treatment up to week 3 and to week 9
    1-5. Change in FIBSER from 1 through 9 weeks
    1-6. Incidence of suicidality as measured with C-CASA between 1 and 9 weeks
    1-7. Incidence of mania, hypomania and mixed episodes between 1 and 9 weeks
    1-8. Incidence of serious adverse events between 1 and 9 weeks.
2. For the acute-phase to continuation treatments,
  (as an index of effectiveness)
    2-1. Proportion of remission (4 or less on PHQ9) at week 25
    2-2. Change in PHQ9 from 1 through 25 weeks
    2-3. Change in BDI-II from 1 through 25 weeks
  (as an index of acceptability and safety)
    2-4. Time to discontinuation of the allocated treatment by week 25
    2-5. Time to discontinuation of any treatment by week 25
    2-6. Change in FIBSER from 1 through 25 weeks
    2-7. Incidence of suicidality as measured with C-CASA between 1 and 25 weeks
    2-8. Incidence of mania, hypomania and mixed episodes between 1 and 25 weeks
    2-9. Incidence of serious adverse events between 1 and 25 weeks

**REPORT #2**

Report #2 will include the following:

1. For the acute phase treatment,
  (as an index of effectiveness)
    1-1. Change in BDI-II from 3 through 9 weeks
    1-2. Proportion of response (50 % or greater reduction from week 1 in PHQ9) at week 9
    1-3. Proportion of remission (four or fewer on PHQ9) at week 9
  (as an index of acceptability and safety)
    1-4. Proportion of successful continuation of the allocated treatment up to week 9
    1-5. Change in FIBSER from 3 through 9 weeks
    1-6. Incidence of suicidality as measured with C-CASA between 3 and 9 weeks
    1-7. Incidence of mania, hypomania and mixed episodes between 3 and 9 weeks
    1-8. Incidence of serious adverse events between 3 and 9 weeks.
2. For the acute-phase to continuation treatments,
  (as an index of effectiveness)
    2-1. Proportion of remission (4 or less on PHQ9) at week 25
    2-2. Change in PHQ9 from 3 through 25 weeks
    2-3. Change in BDI-II from 3 through 25 weeks
  (as an index of acceptability and safety)
    2-4. Time to discontinuation of the allocated treatment by week 25
    2-5. Time to discontinuation of any treatment by week 25
    2-6. Change in FIBSER from 3 through 25 weeks
    2-7. Incidence of suicidality as measured with C-CASA between 3 and 25 weeks
    2-8. Incidence of mania, hypomania and mixed episodes between 3 and 25 weeks
    2-9. Incidence of serious adverse events between 3 and 25 weeks

### Sample size and power

The clinical question for REPORT #2 is the main hypothesis of this trial. Previous studies using PHQ9 in the acute phase treatment of major depression have shown that, on average, the PHQ9 scores will drop from 15 (SD = 5) at baseline to 10 (SD = 6) at the end of treatment, with a mean change of 5 (SD = 5). We expect a difference of 20 % (1 point) in the PHQ9 change scores among the intervention arms and consider this a clinically meaningful difference in effect. With the alpha error set at 0.05 and statistical power at 0.80, in order to detect a between-group difference of 1 point (SD = 5) in the reduction of PHQ9 scores from baseline, we need 522 per group and 1,566 in total for Step II. Assuming a dropout rate of 10 % and a remission rate of 10 % at week 3, we need 1,934 participants at Step I.

The sample size required for REPORT #1 was determined as follows. Assuming an intracluster correlation coefficient to be 0.05 [13, 14], with alpha error at 0.05 and statistical power at 0.80, to detect a difference of 1 point on PHQ9 (SD = 5), that is, to detect an effect size of 0.2 at week 9, we need 66 patients at each of 30 sites. We therefore need 1,980 patients at Step I.

Altogether, we concluded that 2,000 patients would be needed to have enough statistical power to examine our primary outcomes for REPORTS #1 and #2.

### Datasets to be analyzed

The analyses will be performed according to the intention to treat (ITT) principle. The ITT population will consist of all randomized subjects regardless of whether he/she has received the allocated intervention of interest.

### Handling of missing data

We will first tabulate the reasons for missing data, compare the baseline characteristics between participants with missing data versus without missing data, and check the types of mechanism of missingness as follows: completely missing at random, missing at random, and informative missing (Tables 1 and 2).

**Table 1:** Reasons for missing data

	Reasons for missing data
Week 3	Withdrawal of consent to be assessed
	Unable to contact
	….
Week 9	
Week 25

**Table 2:** Comparison of participants with missing data versus participants without missing data

	Participants with missing data at week 3	Participants without missing data at week 3	Statistics
Main demographic and baseline clinical characteristics			
	Participants with missing data at week 9	Participants without missing data at week 9	
Main demographic and baseline clinical characteristics			
	Participants with missing data at week 9	Participants without missing data at week 9	
Main demographic and baseline clinical characteristics			

### Statistical analyses

The draft statistical analysis plan was written by YN, ST and TAF and was subsequently discussed and approved by the Steering Committee. The statistical analyses will be performed by YN and independently confirmed by ST. YN will be responsible for all the analyses. We will use SAS for the analyses.

### Patient flow diagram

The flow of participants will be shown in the Consolidated Standards of Reporting Trials (CONSORT) Flow diagram (Flow diagram for REPORT #1 and REPORT #2).

### Protocol deviations

Participants with protocol deviations in eligibility (for example, when a person with an out-of-range age was erroneously registered) will be excluded from the ITT population, with reasons for exclusion being noted. Those with protocol deviations in treatments and/or assessments will be tabulated (Tables 3 and 4) and will be included in the ITT analysis.

**Table 3:** Protocol deviations

A. Treatment adherence	Total
	N (%)
A1. In sertraline 100 mg group, 100 mg not achieved at week 3	
A2. In mirtazapine group, sertraline still used (no zero) at week 7.

**Table 4:** Protocol deviations

	Step1		Step2				Step3
	Weeks 1 to 3		Weeks 3 to 9				Weeks 9 to 25
	50 mg	100 mg	Continue sertraline	Augment	Switch	Remitted	
	N (%)	N (%)	N (%)	N (%)	N (%)	N (%)	N (%)
B. Treatment deviation							
B1. Prohibited concurrent treatments							
B2. Impossible to randomize at week 3							
B3. No tolerability to sertraline at step 1							
B4. Mania at week 1 though week 3							
B5. Mania or diagnosis of schizophrenia or dementia at week 3 through 25							
C. Stopping intervention and assessment							
C1. The participant wishes to stop the protocol treatment.							
C2. The trial physician judges that it is difficult to continue the protocol treatment because of the emergence of serious adverse events (SAE) as defined below.							
C3. The trial physician judges that the risk outweighs the benefit in continuing the protocol treatment even when no SAE is reported.							
C4. The participant becomes pregnant and the trial physician judges that the risk outweighs the benefit in continuing the protocol treatment.							
C5. The trial physician judges that it is inappropriate to continue the protocol treatment for any other reason.							
D.							
D1. The participant withdraws consent to receiving protocol assessments, regardless of whether he/she is continuing the protocol treatment.							
E.							
E1. Violation of allowance in timing of assessments of outcomes							

### Demographic and baseline clinical characteristics of participants

Demographic characteristics of all participants will be described per allocated arm in a table describing the following variables: age, sex (woman or man), education (years), job (employed full-time, employed part-time, on sick leave, housewife, student or no employment), and marriage (single, never married; single, divorced or separated; single, deceased; or married). Clinical characteristics to be presented will include the following: age of onset at first episode, number of previous depressive episodes, length of current episode, out- or inpatient status at time of entry into the study, PHQ9 at baseline, BDI-II at baseline, and physical comorbidities.

Continuous variables will be presented as mean and standard deviation or, if a considerable skew is present, as median and interquartile range. The maximum and the minimum of the reported values will also be noted. Binary and categorical variables will be presented as the number of participants and percentage. The *P* values will be calculated for comparisons, if necessary (Tables 5 and 6).

**Table 5:** Demographic and baseline clinical characteristics for REPORT #1

	50 mg/d arm	100 mg/d arm
Demographic		
Age, mean (SD), min-max		
Sex, n (%)		
Education n (%)		
Junior High school		
….		
Job n (%)		
Employed full time		
….		
Marriage n (%)		
Single, never married		
Clinical		
Age of onset at first episode, years,		
Number of previous depressive episodes, N (%)		
Length of current episode, months		
Out- or inpatient status at time of entry, N (%)		
PHQ9 at time 0,		
Mean (SD), range		
PHQ9 at baseline,		
Mean (SD), range		
BDI at baseline,		
Mean (SD), range		
Physical conditions, N (%)		
No physical comorbidity		
…..

**Table 6:** Demographic and baseline characteristics for REPORT #2

	Sertraline continuation	Mirtazapine augmentation	Mirtazapine switch
Demographic			
Age, mean (SD)			
Sex, n (%)			
Education n (%)			
Junior High school			
….			
Job n (%)			
Employed full time			
….			
Marriage n (%)			
Single, never married			
….			
Clinical			
Age of onset at first episode, years,			
Number of previous depressive episodes, N (%)			
Length of current episode, months			
Out- or inpatient status at time of entry, N (%)			
PHQ9 at time 0,			
Mean (SD), range			
PHQ9 at baseline,			
Mean (SD), range			
BDI at baseline,			
Mean (SD), range			
Physical conditions, N (%)			
No physical comorbidity			
….			

### Treatments received

The kinds and amounts of the protocol treatments actually prescribed will be summarized for each intervention arm at the following time points: week 0; previous day of weeks 1, 2, 3, 4, 5, 6, 7, 8, and 9 and previous day of weeks 13, 17, 21 and 25 (Tables 7 and 8).

**Table 7:** Treatments received for each intervention for REPORT #1

	50 mg/d arm	100 mg/d arm
Week 0		
Previous day of Week 1		
Previous day of Week 2		
Previous day of Week 3		
Previous day of Week 4		
Previous day of Week 5		
Previous day of Week 6		
Previous day of Week 7		
Previous day of Week 8		
Previous day of Week 9		
Previous day of Week 13		
Previous day of Week 17		
Previous day of Week 21		
Previous day of Week 25

**Table 8:** Treatments received for each intervention for REPORT #2

	Sertraline continuation	Mirtazapine augmentation	Mirtazapine switch
Previous day of Week 4			
Previous day of Week 5			
Previous day of Week 6			
Previous day of Week 7			
Previous day of Week 8			
Previous day of Week 9			
Previous day of Week 13			
Previous day of Week 17			
Previous day of Week 21			
Previous day of Week 25			

### Analyses for the primary outcome

For the primary outcome in REPORT #1, we will use a mixed-model repeated-measures analysis to compare model-adjusted least-squares means of PHQ-9 at 9 weeks. The model will include the fixed effects of PHQ-9 score at week 1; all demographic and baseline clinical variables with statistically significant imbalance between the two arms; treatment (sertraline 50 mg/d versus 100 mg/d); visit and treatment-by-visit interaction; and clinic/hospital size (whether each site could be expected to recruit 40 patients or more per year, a condition used as a stratification variable in cluster randomization). The random effect will include the participant and the cluster. Study visit will be treated as a categorical variable.

The population of interest in this comparison is all the randomized population under continued/intended sertraline treatment, including those who have dropped out of the protocol treatment by week 3, those who have remitted by week 3 (both of whom were therefore not randomized for Step II), and one random third of the remaining patients who have been randomized to continue on sertraline for Step II (blue-shaded cells in Fig. 1). In other words, the outcome data will be missing for patients who have been randomized to mirtazapine augmentation or mirtazapine switching for Step II. We will therefore use inverse probability of censoring weighting to account for the presence of missing data in parameter estimation for the mixed-models repeated-measures.

We will present parameters estimated from the model as well as the effect sizes between the treatments as mean difference (MD) and standardized mean difference (SMD); in order to calculate SMD, SD of the completers’ endpoint scores will be used (Table 9). We will illustrate the estimated and actual average courses through the acute and continuation treatment phases by interventions (Figure and average course).

**Table 9:** Outcomes for REPORT #1

	50 mg/d arm, Mean (SD) n (%)	100 mg/d arm, Mean (SD) n (%)	Difference, RR, HR (95 % CI), *P* value, and adjusted 1, 2,..	*P* value
Primary				
Secondary 1				
Secondary 2				
Secondary 3				
Secondary 4				
…..				
Secondary 16				
Item 1				
…				
(Subgroup)				
XXXX				
Primary				
Secondary				
….				

For the primary outcome in REPORT #2, we will examine whether the changes in PHQ9 scores from week 3 through week 9 are statistically significantly different among the sertraline continuation arm, the mirtazapine augmentation arm and the mirtazapine switch arm. We will use a mixed-model repeated-measures analysis to compare model-adjusted least-squares means of PHQ-9 at 9 weeks. The model will include the fixed effects of PHQ-9 score at week 3; treatment (sertraline continuation versus mirtazapine augmentation versus mirtazapine switch); visit and treatment-by-visit interaction; stratification variables ((i) site, (ii) whether 50 % or greater reduction on PHQ9 is achieved or not, and (iii) whether “moderate” or greater impairment due to side effects is reported on item 4 of FIBSER); and the Step I treatment (sertraline 50 mg/d versus 100 mg/d). The random effect will be the participant. Study visit will be treated as a categorical variable. We will examine the interaction effect of Step I randomization by adding the Step II treatment by Step I treatment interaction term in the above model.

We will present parameters estimated from the model as well as the effect sizes between the treatments as mean difference (MD) and standardized mean difference (SMD): in order to calculate SMD, SD of the completers’ endpoint scores will be used (Table 10. ). We will illustrate the estimated and actual average courses through the acute and continuation treatment phases by interventions (Figure and average course).

**Table 10:** Outcomes for REPORT #2

	Sertraline continuation	Mirtazapine augmentation	Mirtazapine switch	Difference, RR, HR (95 % CI), *P* value, and adjusted 1, 2,	Difference	Difference
Primary PHQ9 total score, mean, (SD, 95 % CI)						
Effect size						
Model-based coefficient, *P* value						
Secondary1 FIBSER total score, mean, (SD, 95 % CI)						
Effect size						
Model-based coefficient, *P* value						
Secondary 2						
…						
Secondary 16						
Item 1 n (%)						
….						
(Subgroup)						
XXXX						
Primary						
Secondary						
….						

### Analyses for the secondary outcomes

We will perform secondary analyses to supplement our primary analyses and obtain more detailed understanding of our clinical questions. The secondary analyses will use models similar to those of the primary analyses. We will calculate mean difference and their 95 % CI for differences for continuous outcomes and relative risks (RR) and their 95 % CI for dichotomous outcomes.

We will calculate hazard ratios (HR) in Cox regression and their 95 % CI for differences in continuation of the allocated treatment and of any treatment. Cases who withdrew consent to assessment and cases lost to follow-up (for example, moving away) will be censored. In survival analysis, we will check the assumptions of proportional hazard (Tables 9 and 10 ) (Figure, Average course and survival curve).

### Subgroup analyses

For REPORT #1, we will conduct the following a priori specified subgroup analyses: (i) whether the baseline PHQ9 score was 15 or more (moderate to severe depression) or not, and (ii) whether the patient had shown greater or smaller improvement from week 0 to week 1 on sertraline 25 mg/d (split at the median of the observed improvements).

We will analyze subgroups for REPORT #2 according to the following stratification factors used for randomization at Step II: (i) whether 50 % or greater reduction on PHQ9 was achieved from week 1 to week 3 or not, and (ii) whether “moderate” or greater impairment due to side effects was reported on item 4 of FIBSER at week 3, as well as (iii) which treatment arm of Step I the patient was on.

### Sensitivity analyses

For the primary outcomes in REPORTS #1 and #2, we will conduct the following sensitivity analyses:

1. We will conduct completers’ analyses instead of ITT analyses.
2. We will conduct the same mixed-model repeated-measures analyses but with visit included as a continuous variable. For this analysis, two models will be compared in which the visit timing is treated as a linear variable or as a log-transformed variable, and their respective model fit index will be reported.
3. We will also conduct the same mixed-model repeated-measures analyses with the actual assessment timing, not the scheduled assessment timing, for the above two models. Their respective model fit index will also be reported.

For the primary outcome in REPORT #1 we will further conduct the following sensitivity analyses.

4. For REPORT #1, we will use analysis of covariance (ANCOVA) without within-clustered correlation instead of the mixed effect model with within-cluster correlations, in order to check the assumption of statistical property by the simpler analysis, which do not need such assumptions.
5. For REPORT #1, we will conduct the mixed-model repeated-measures analyses without covariates, that is, without demographic and baseline clinical variables that have shown statistically significant imbalance between the two arms.

### Multiplicity

For each of REPORTS #1 and #2, the significance level will be set at *P* < 0.05. All statistical testing will be two-sided. In REPORT #2, we will compare three arms and will use Hochberg method for adjustment of multiplicity [15] in the primary analysis. Only analyses for the primary outcomes have confirmatory nature and will be controlled for multiplicity. Secondary analyses and secondary outcomes will not be adjusted for multiplicity, because these analyses have explanatory and supplemental nature for the primary analyses.

### Evaluation of blinding of outcome assessment

We will calculate kappa coefficients in treatment guesses for week 3, 9 and 25 assessments in order to ascertain the assessors’ blindness to the allocated treatments (Tables 11 and 12).

**Table 11:** Blinding at week 3

Actual	50 mg/d	100 mg/d
Guess		
50 mg/day at week 3		
100 mg/day at week 3		
Continue sertraline at weeks 9 or 25		
Augment with mirtazapine at weeks 9 or 25		
Switch to mirtazapine at weeks 9 or 25		
Remitted at weeks 9 or 25

**Table 12:** Blinding at weeks 9 and 25

Actual	Continue sertraline	Augment	Switch	Remitted
Guess				
50 mg/day at week 3				
100 mg/day at week 3				
Continue sertraline at weeks 9 or 25				
Augment with mirtazapine at weeks 9 or 25				
Switch to mirtazapine at weeks 9 or 25				
Remitted at weeks 9 or 25				

### Blind interpretation committee

The statistical analysis report will present the primary results for REPORTS #1 and #2 without breaking the randomization to the writing committee. The writing committee will develop interpretations of the results and associated conclusions assuming for REPORT #1 that Treatment A is the lower dose, and then assuming Treatment B is the lower dose. Similarly, for REPORT #2, the interpretations will be developed assuming that Treatment A is any one of the three possibilities, as with Treatment B and C. These interpretations will be recorded, signed by the participants and made public on the study web site at that time. The randomization code will then be broken, the correct interpretation will be chosen, and the manuscript will be finalized accordingly [16, 17].

### Summary of changes from the original protocol

In the original protocol [1], we had specified the following primary and secondary outcomes for each of Steps I through III.

**Table:** 

Step I
Patients: Patients with non-psychotic unipolar major depressive episode who had not received treatment for the index episode before starting sertraline and who tolerate sertraline at 25 mg/d
Exposure 1: Strategy to titrate sertraline up to the maximum of the effective range, that is, 25 mg/d ≥ 50 mg/d ≥ 100 mg/d
Exposure 2: Strategy to titrate sertraline up to the minimum of the effective range, that is, 25 mg/d ≥ 50 mg/d ≥ 50 mg/d
Outcomes: The primary outcome is the change in PHQ9 scores at week 1 through week 3
The secondary outcomes include:
Change in BDI2 scores at week 1 through week 3
Proportion of remission (four fewer on PHQ9) at week 3
Proportion of response (50 % or greater reduction on PHQ9) at week 3
Proportion of successful continuation of the allocated treatment up to week 3
Change in FIBSER at week 1 through week 3
Change in PHQ9 at week 1 through week 9
Change in BDI2 at week 1 through week 9
Proportion of remission (four or fewer on PHQ9) at week 9
Proportion of response (50 % or greater reduction on PHQ9) at week 9
Proportion of successful continuation of the allocated treatment up to week 9
Change in FIBSER at week 1 through week 9
Suicidality as assessed with C-CASA between week 1 and week 9
Manic/hypomanic/mixed episode between week 1 and week 9
Serious adverse events between week 1 and week 9
Step II
Patients: Patients whose major depressive episode did not remit (five or more on PHQ9) at week 3 to the first-line treatment with sertraline
Exposure 1: Continue sertraline 50 mg/d or 100 mg/d for 6 more weeks
Exposure 2: Augment sertraline with mirtazapine 15 to 45 mg/d
Exposure 3: Switch to mirtazapine 15 to 45 mg/d
Outcome: The primary outcome is the change in PHQ9 at week 4 through week 9
The secondary outcomes include:
Change in BDI2 at week 4 through week 9
Proportion of remission (four or fewer on PHQ9) at week 9
Proportion of response (50 % or greater reduction on PHQ9) at week 9
Proportion of successful continuation of the allocated treatment up to week 9
Change in FIBSER at week 4 through week 9
Suicidality as assessed with C-CASA between week 3 and week 9
Manic/hypomanic/mixed episode between week 3 and week 9
Serious adverse events between week 3 and week 9
Step IIIa (exploratory analysis of continuation treatment for Step I)
Patients: Patients with nonpsychotic unipolar major depressive episode who had not received treatment for the index episode before starting sertraline and who tolerate sertraline 25 mg/d
Exposure 1: Strategy to titrate sertraline up to the maximum of the effective range, that is, 25 mg/d ≥ 50 mg/d ≥ 100 mg/d by week 3, then allocated to continue sertraline between week 3 and week 9, then treated at the discretion of the trial physician
Exposure 2: Strategy to titrate sertraline up to the minimum of the effective range, that is, 25 mg/d ≥ 50 mg/d ≥ 50 mg/d by week 3, then allocated to continue sertraline between week 3 and week 9, then treated at the discretion of the trial physician
Outcome: The primary outcome is the proportion of patients who continue the allocated treatment up to week 25 and are in remission (4 or less on PHQ9) at week 25
The secondary outcomes include:
Proportion of patients who continue the allocated treatment up to week 25 and are showing response (50 % or greater reduction on PHQ9) at week 25
Rate of continuation of allocated treatments up to week 25
Change in PHQ9 at week 1 through week 25
Change in BDI2 at week 1 through week 25
Suicidality as assessed with C-CASA between week 1 and week 25
Manic/hypomanic/mixed episode between week 1 and week 25
Serious adverse events between week 1 and week 25
Step IIIb (exploratory analysis of continuation treatment for Step II)
Patients: Patients whose major depressive episode did not remit (five or more on PHQ9) at week 3 to the first-line treatment with sertraline
Exposure 1: Continue sertraline 50 mg/d or 100 mg/d for 6 more weeks, then treated at the discretion of the trial physician
Exposure 2: Augment sertraline with mirtazapine 15 to 45 mg/d up to week 9, then treated at the discretion of the trial physician
Exposure 3: Switch to mirtazapine 15 to 45 mg/d up to week 9, then treated at the discretion of the trial physician
Outcome: The primary outcome is the proportion of patients who continue the allocated treatment up to week 25 and are in remission (four or fewer on PHQ9) at week 25
The secondary outcomes include:
Proportion of patients who continue the allocated treatment up to week 25 and are showing response (50 % or greater reduction on PHQ9) at week 25
Rate of continuation of allocated treatments up to week 25
Change in PHQ9 at week 4 through week 25
Change in BDI2 at week 4 through week 25
Suicidality as assessed with C-CASA between week 3 and week 25
Manic/hypomanic/mixed episode between week 3 and week 25
Serious adverse events between week 3 and week 25

In this updated protocol, we have merged Step I with Step IIIa and Step II with Step IIIb into two main reports in order to present the results of this complex trial in the most clinically informative way. These two reports will focus on Step I randomization and Step II randomization, respectively, and both will focus on the acute phase treatment up to 9 weeks as their primary outcome but supplement this with other benefit and harm outcomes for the acute phase treatment, as well with benefit and harm outcomes for the continuation treatment up to 25 weeks.

REPORT #1 will examine the clinical questions for Step I through Step IIIa, as originally postulated in the published protocol. For this report, however, we have changed the primary outcome from “Change in PHQ9 from 1 through 3 weeks” to “Change in PHQ9 from 1 through 9 weeks” (which was one of the secondary outcomes in the original protocol), in order to fully examine our clinical question comparing the 50 mg/day arm and 100 mg/day arm up to the end of the acute treatment phase. Week 9 was chosen as the primary outcome because the acute phase treatment usually is considered to last 6 to 12 weeks [2, 3]. In order to focus on the effect of the choice of the initial dosage of the first-line treatment with sertraline through the acute treatment phase as defined, we will concentrate on those who did not remit and were subsequently randomized at week 3 to continue on sertraline (hence, a random third of those who had not remitted by week 3), those who had remitted and were therefore continued on sertraline, and those who had dropped out of the protocol treatment by week 3 and subsequently received treatments at physician’s discretion, in order to abide by the ITT principle for Step I randomization (see blue cells in Fig. 1).

REPORT #2 will deal with the clinical question for Step II and Step IIIb, as originally postulated in the published protocol, and has set the primary outcome for Step II as its primary outcome.

The required sample size at Step I remained the same for REPORT #1 by assuming the same magnitude to effect to detect at week 9 instead of at week 3. The sample size required at Step II for REPORT #2 also remained the same; however, we reduced the initial sample size to be recruited into Step I in order to ensure this sample after we reviewed the observed proportion of patients who had entered into Step I but subsequently dropped out of the protocol treatment and could not be randomized at Step II (from 20 % to 10 %).

All these changes were discussed and agreed upon by the Steering Committee of the SUN(^_^)D trial before the randomization code is broken.

## Conclusion

This paper presents the updated protocol and the detailed statistical analysis plan for the SUN(^_^)D trial in order to avoid risks of reporting bias and data-driven results. Due to the complexity of the trial, we plan to report the main results of the trial in two reports, and this updated protocol and statistical analysis plan indicate the respective primary and secondary outcomes and their analyses.

## Abbreviations

BDI-II: Beck Depression Inventory, 2nd edition
C-CASA: Columbia Classification Algorithm for Suicide Assessment
FIBSER: Frequency, Intensity and Burden of Side Effects Rating
PHQ9: Personal Health Questionnaire-9
PRIME-MD: Primary Care Evaluation of Mental Disorders
SUN(^_^)D: Strategic Use of New Generation Antidepressants for Depression
